# Impact of the carbon flux regulator protein *pirC* on ethanol production in engineered cyanobacteria

**DOI:** 10.3389/fmicb.2023.1238737

**Published:** 2023-08-15

**Authors:** Julien Böhm, Karsten Kauss, Klaudia Michl, Lisa Engelhardt, Eva-Maria Brouwer, Martin Hagemann

**Affiliations:** ^1^Department Plant Physiology, Institute of Biosciences, University of Rostock, Rostock, Germany; ^2^Department Aquatic Ecology, Institute of Biosciences, University of Rostock, Rostock, Germany; ^3^Department Microbiology, Institute of Biosciences, University of Rostock, Rostock, Germany

**Keywords:** biofuel, carbon availability, CO_2_, green biotechnology, nitrogen limitation

## Abstract

Future sustainable energy production can be achieved using mass cultures of photoautotrophic microorganisms such as cyanobacteria, which are engineered to synthesize valuable products directly from CO_2_ and sunlight. For example, strains of the model organism *Synechocystis* sp. PCC 6803 have been generated to produce ethanol. Here, we performed a study to prove the hypothesis that carbon flux in the direction of pyruvate is one bottleneck to achieve high ethanol titers in cyanobacteria. Ethanol-producing strains of the cyanobacterium *Synechocystis* sp. PCC 6803 were generated that bear mutation in the gene *pirC* aiming to increase carbon flux towards pyruvate. The strains were cultivated at different nitrogen or carbon conditions and the ethanol production was analysed. Generally, a clear correlation between growth rate and ethanol production was found. The mutation of *pirC*, however, had only a positive impact on ethanol titers under nitrogen depletion. The increase in ethanol was accompanied by elevated pyruvate and lowered glycogen levels indicating that the absence of *pirC* indeed increased carbon partitioning towards lower glycolysis. Metabolome analysis revealed that this change in carbon flow had also a marked impact on the overall primary metabolism in *Synechocystis* sp. PCC 6803. Deletion of *pirC* improved ethanol production under specific conditions supporting the notion that a better understanding of regulatory mechanisms involved in cyanobacterial carbon partitioning is needed to engineer more productive cyanobacterial strains.

## Introduction

The present day increase of CO_2_ in the atmosphere due to human activities is one of the main drivers of the global climate change (IPCC, 2021). To minimize the negative impact on our societies, the combustion of fossil carbon sources needs to be reduced and should be replaced by sustainable processes using CO_2_ as carbon source. Oxygenic photosynthesis, the carbon assimilation pathway performed by photoautotrophic organisms represents an environmental friendly possibility to produce food and feedstock by using atmospheric CO_2_ and light energy. Cyanobacteria are the only prokaryotic organisms that perform this pathway for the generation of biomass. In contrast to plants and eukaryotic algae, these organisms are more easily accessible for genetic manipulations making it possible to plug in desired pathways for the production of biofuels or valuable feedstock [reviewed in Hagemann and Hess (2018) and Liu et al. (2021)]. Such engineered cyanobacterial strains are regarded as green cell factories, which can be applied in a sustainable CO_2_-neutral biotechnology harnessing solar energy. Mass cultivation of cyanobacteria can be performed on non-arable land thereby minimizing the competition with agricultural food production. Moreover, many cyanobacteria can tolerate high salinities, which permits the use of brackish or marine water for their mass cultivation and reduces dependence on limiting freshwater resources (Chisti, 2013; Pade and Hagemann, 2015; Pade et al., 2017). These features make cyanobacteria a promising engineerable chassis for the future sustainable bioeconomy, however, the present strains usually show only low productivities making their application infeasible.

The cyanobacteria-based production of the biofuel ethanol is one example, which is commercially promising but is still limited by rather low productivities compared to traditional ethanol production by yeast using plant-derived organic carbon as precursor (Andrews et al., 2021). In fact, the generation of an ethanol-producing *Synechococcus elongatus* strain by expression of pyruvate decarboxylase (PDC) and ethanol dehydrogenase (ADH) from *Zymomonas mobilis* was one of the first examples to generate a biofuel-releasing cyanobacterium (Deng and Coleman, 1999). Later on, it turned out that the native ADH from cyanobacteria, e.g., *Synechocystis* sp. PCC 6803 provided better features, particularly because it uses NADPH_2_ as the dominating reducing agent in photosynthetic cyanobacterial cells (Dienst et al., 2014). In order to improve ethanol production, several cyanobacterial strains have been engineered, which show different growth rates and resistances towards environmental stresses [reviewed in Andrews et al. (2021)].

However, preliminary work indicated that the production of ethanol in engineered cyanobacterial strains depends on the amount of the precursor pyruvate (Kopka et al., 2017). Recently, it has been shown that the deletion of the regulator protein *pirC* resulted in a significant accumulation of pyruvate in N-limited cells of *Synechocystis* sp. PCC 6803, because *pirC* can inhibit the phosphoglycerate mutase and thereby the carbon partitioning into lower glycolysis (Orthwein et al., 2021). This has been particularly observed under N-limiting conditions when the regulatory metabolite 2-oxoglutarate accumulates, which releases *pirC* from its PII-bound state and initiates strong *pirC*-mediated phosphoglycerate mutase inactivation (Orthwein et al., 2021). Therefore, we expected that engineering of ethanol production in the background of mutant Δ*pirC* should result in higher ethanol titers particularly under low nitrogen conditions when increased amounts of pyruvate are available. To prove this hypothesis, an ethanol-producing strain of the cyanobacterium *Synechocystis* sp. PCC 6803 was generated that bears mutation in the gene *pirC*. The ethanol production of this strain was compared to an ethanol-producing *Synechocystis* sp. PCC 6803 wild-type (WT) strain when cultivated under different conditions.

## Material and methods

### Generation and characterization of strains

The glucose-sensitive *Synechocystis* sp. PCC 6803 strain was used as wild type (WT) in the entire study. The generation of the *pirC* deletion mutant (Δ*pirC* containing a kanamycin-resistance cassette) has been described in Orthwein et al. (2021). To generate ethanol-producing strains of the WT and the mutant Δ*pirC*, the ethanologenic cassette 219 (received from Dr. Dan Kramer, Cyano Biotech Berlin, Germany) was transferred into the cyanobacterial cells via conjugation. The ethanologenic cassette 219 expresses the *pdc* from *Z. mobilis* and the *adh* from *Synechocystis* sp. PCC 6803 under control of the copper-regulated promoter P*_petJ_* on the autonomous-replicating plasmid pVZ325. It has been shown that this plasmid permits a fourfold higher expression of the *adh* gene under copper-free cultivation conditions compared to WT (Dienst et al., 2014). Conjugants receiving the cassette 219 were selected on gentamycin. All strains used in the present study are listed in Supplementary Table S1.

To verify the genotypes of the different strains, DNA was isolated from the cyanobacterial suspensions. PCR reactions were performed with primers specific for the *pirC* or *pdc* genes as listed in Supplementary Table S2. The PCR revealed that all strains had the correct genotypes as shown in the Supplementary Figure S1.

### Cultivation of strains

All strains were pre-cultured on plates containing BG11 and 0.8% bacto-agar for 1 week and were subsequently transferred in Erlenmeyer flasks with BG-11 medium (Rippka et al., 1979; TES pH 8.0) for another week. To induce the “ethanol-producing” genes, the cultures were then grown in BG-11 medium without copper for 1 week. The initial screening of ethanol production was performed in shaken Erlenmeyer flasks containing 50 mL cyanobacterial suspension at 30°C and white light of 50 μmol photons m^−2^ s^−1^. The ethanol production assays were conducted in batch cultures aerated with CO_2_-enriched air (5%, v/v) in the Multi-Cultivator MC 1000-OD (PSI, Czech Republic) at 30°C. The culture volume was 60 mL. Three experimental conditions were tested: (1) 250 μmol photons m^−2^ s^−1^, BG-11 without copper; (2) 500 μmol photons m^−2^ s^−1^, BG-11 without copper; (3) 500 μmol photons m^−2^ s^−1^, BG-11 with ten-times reduced nitrate amounts (final concentration 1.8 mM NaNO_3_, while the original BG11 medium contains 17.65 mM NaNO_3_) and without copper. When cells were transferred into media with reduced nitrogen contents, the cell pellets were washed one-time with BG11 medium containing either no or one-tenth nitrate. The culture media contained the appropriate antibiotics. Growth was continuously followed by measuring the optical density at 720 nm (OD_720_) in the Multi-Cultivator MC1000-OD and upon sampling the OD_720_ was determined manually with a photometer on days 0, 3, 5, and 7. Loss of volatile ethanol from the cultivation vessels due to aeration was quantified by sparking the outflowing gas stream into collecting bottles filled with either 300 or 600 mL distilled water. The dissolved ethanol in the collecting vessels was related to the volume of the cultures. Samples for ethanol quantification were taken on days 3, 5, and 7 from the cultures. Samples for metabolite quantification via LC-MS/MS and glycogen analysis were taken on day 7. A few experiments were conducted for longer times.

### Ethanol quantification

For ethanol quantification, 2 mL samples were taken from each culture. These were centrifuged three-times (10 min, 14,000 g, 4°C). After each centrifugation step, the supernatant was transferred into a new vial. The final supernatants were then used for ethanol quantification via GC. Initially, ethanol was enzymatically determined in the cyanobacterial suspension, whereby ethanol was oxidized to acetaldehyde by ADH in the presence of NAD^+^. As done previously (Pade et al., 2017), we used the Ethanol-Test-Kit (r-biopharm Enzytec, Darmstadt, Germany) according to the manufacturer’s protocol. Instead of using a reaction volume of 3 mL, the assay was performed in a total volume of 0.2 mL keeping the same molar ratios of all reagents. The ethanol quantification was performed in 96-well microplates at room temperature using the plate-reader Synergy HTX (BioTek, United States). Ethanol concentration were calculated using an ethanol calibration curve. Furthermore, we used the kit ab272531 (Abcam, United States), which is based on the dichromate method. Here, we proceeded exactly according to the manufacturer’s protocol.

In the main experiments, ethanol was quantified using gas-chromatography (GC), because this method produced more reliable data (see below). For the GC analysis, 500 μL sterile H_2_O, 50 μL phosphoric acid (2 M), and 450 μL sample were mixed in a vial and sealed. Three technical replicates were prepared for each biological sample. A gas chromatograph (8860 GC System, Agilent Technologies, United States) was used for the analysis. For the calculation of the absolute ethanol concentrations, a calibration was done in advance. All ethanol amounts were related to total culture volume or to OD_720_ of the cyanobacterial suspension as proxy for biomass.

### Glycogen quantification

Cellular glycogen content was determined as described by Lucius et al. (2021). Three 4 mL aliquots of cells were harvested at the end of each experiment. The washed pellets were suspended in 400 μL KOH (30% w/v) and then incubated at 95°C for 2 h. Glycogen was precipitated with 1.2 mL of 96% EtOH at −20°C overnight. The washed pellets were re-suspended in 1 mL sodium acetate buffer (100 mM, pH 4.5) with 100 μL of amyloglucosidase mix (0.6 mL amyloglucosidae Sigma/Aldrich mixed with 5 mL Na-acetate buffer). The samples with amyloglucosidase mix were incubated for 2 h at 37°C. The produced glucose was stained with 250 μL o-toluidin reagent at 100°C for 10 min. Glucose contents were calculated using a glucose calibration curve.

### Metabolite quantification via LC-MS/MS

The metabolite analysis was carried out as described in Reinholdt et al. (2019), whereby the extraction was modified. At the end of the experiment, cells were harvested in triplicates of 0.7–2 mL (depending on the experimental conditions) on nitrocellulose filters (25 mm, Porafil, Macherey-Nagel) and immediately frozen in liquid nitrogen. The filter with cell material was mixed with 630 μL methanol in the frozen state and 1 μL carnitine (1 mg mL^−1^) was added as an internal standard. The samples were vortexed for 1 min, incubated in an ultrasonic bath for 5 min and shaken for 15 min. Subsequently, 400 μL of 99.9% chloroform was added and the samples were incubated for 10 min at 37°C, followed by addition of 800 μL H_2_0. The samples were then incubated overnight at −20°C for improved precipitation. The samples were centrifuged (5 min, 14,000 rpm, 4°C) and the upper polar phase was transferred into a new 2 mL centrifuge tube. After drying in a vacuum centrifuge, the pellets were dissolved in 700 μL H_2_0, shaken for 30 min, and passed through 0.2 μm filters (Omnifix^®^-F, Braun, Germany). Cleared supernatants were analyzed using the high-performance liquid chromatograph mass spectrometer system (LCMS-8050, Shimadzu, Japan) on a pentafluorophenylpropyl (PFPP) column (Supelco Discovery HS FS, 3 μm, 150 × 2.1 mm). Aliquots were continuously injected in the MS/MS part and ionized via electrospray ionization (ESI). The compounds were identified and quantified using the multiple reaction monitoring (MRM) values given in the LC-MS/MS method package and the LabSolutions software package (Shimadzu, Japan).

### Statistical evaluation

All experiments were conducted with at least two biological replicates. All chemical determinations were done in triplicates. The statistical analysis and graphical representation of the data were done in RStudio (Posit PBC, United States).

## Results

### Experiments to identify optimized cultivation and ethanol detection methods

Preliminary work indicated that the production of ethanol in engineered cyanobacterial strains depends on the amount of the precursor pyruvate (Kopka et al., 2017), which has been found at elevated levels after deletion of the regulator protein *pirC* in nitrogen-limited cells of *Synechocystis* sp. PCC 6803 (Orthwein et al., 2021). Therefore, we expected that engineering of ethanol production in the background of mutant Δ*pirC* should result in higher ethanol titers particularly under low nitrogen conditions. To prove this hypothesis, the mutant Δ*pirC* and the corresponding WT were conjugated with a plasmid containing the ethanologenic cassette 219, which expresses the pyruvate decarboxylase (*pdh*) from *Z. mobilis* and the ethanol dehydrogenase (*adh*) from *Synechocystis* sp. PCC 6803 under control of a copper-regulated promoter (Dienst et al., 2014). Prior to experiments, the genotypes of all strains were verified by PCR reactions, which revealed the inactivation of the *pirC* gene in the mutant Δ*pirC* and the presence of the “ethanol-producing” cassette 219 in the corresponding strains (Supplementary Figure S1).

In preliminary experiments, these strains were cultivated in shaken flasks under standard or nitrogen-free conditions, i.e., in nitrate-replete or nitrate-deplete BG11 medium at ambient air. Cells without the cassette 219 produced only small amounts of ethanol close to the detection limit of the ethanol detection kit regardless of the available nitrogen amounts, whereas strains harboring the cassette 219 produced higher ethanol amounts that were slightly elevated under nitrogen-free conditions (Supplementary Figure S2). However, the overall amount of ethanol was rather low. Especially under N-free conditions, the final ethanol titers were already reached after 4 days, thereafter, ethanol yield started to decline. Measurements of OD_750_ as proxy for growth revealed that the cells showed only slow growth under these conditions especially in the absence of the nitrogen source (data not shown).

**Figure 1:**
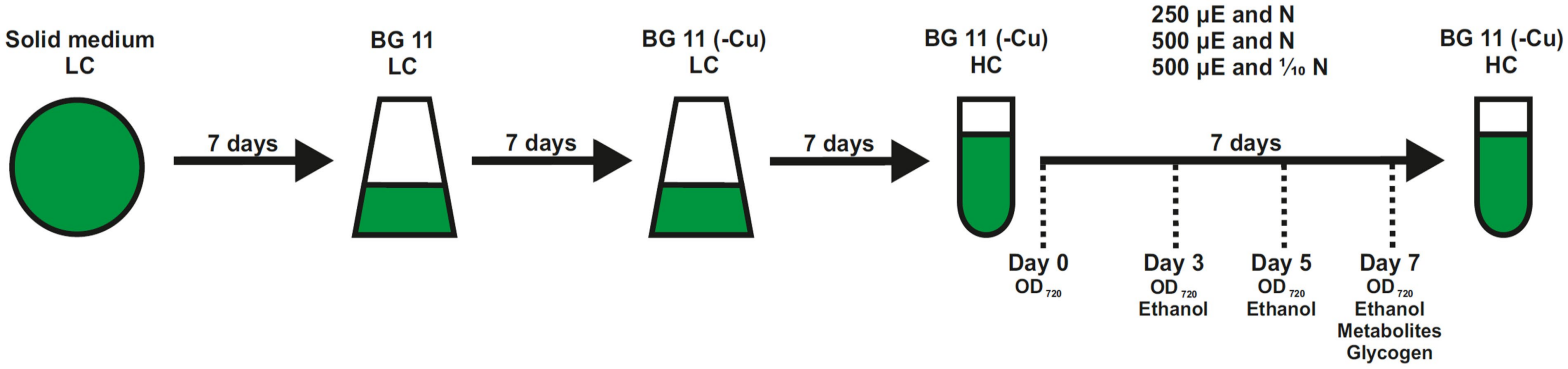
Experimental setup to evaluate the ethanol production in different strains of *Synechocystis* sp. PCC 6803. All strains were maintained on agar plates. Cells were freshly suspended into Erlenmeyer flasks and pre-cultured shaking at 50 μmol photons m^−2^ s^−1^ (μE) in complete BG11 under ambient air (0.04%CO_2_, LC) for 1 week. Then, cells were grown in copper-free (-Cu) BG11 to induce expression of the ethanologenic cassette. Production assays were done in the Multi-Cultivator MD1000 in BG11-Cu at high CO_2_ (5%, HC) and different light intensities of 250 or 500 μmol photons m^−2^ s^−1^ for 1 week. Sampling time points are indicated.

From these experiments we concluded that nitrogen limitation might have a small stimulatory effect on ethanol production, however, the limited growth of the cells prevents higher ethanol titers in this cultivation mode. Accordingly, we then conducted experiments in which bicarbonate was added during the cultivation in shaken Erlenmeyer flasks to stimulate growth and to induce an increased carbon/nitrogen ratio. It has been shown that high CO_2_ (5% CO_2_) conditions decrease the abundance of *pirC* in the *Synechocystis* sp. PCC 6803 WT (Spät et al., 2021). As expected, the addition of bicarbonate stimulated the growth by roughly 50% and the ethanol titers increased to a similar extent, but remained rather low (data not shown). Collectively, these data indicated that growth and ethanol production seem to be closely connected. Hence, all further experiments were conducted in batch cultures that were aerated with CO_2_-enriched air (5% CO_2_, v/v) to permit faster growth and higher cell densities (Figure 1).

Initially, we performed the ethanol quantification using an enzymatic assay, in which ethanol was detected by NAD^+^ reduction via ADH. However, this method displayed unexpected high background values with WT cells not expressing the cassette 219 and resulted in high standard deviation among the technical replicates (see Supplementary Figure S2). Therefore, we included a validation of different ethanol quantification methods into our study. Two different photometric kits (suppliers r-biopharm Enzytec and Abcam) were compared with direct ethanol quantification by gas chromatography (GC). The three methods resulted in quite different ethanol amounts in cell suspensions from the same ethanol production experiment (Supplementary Figure S3), however, they displayed principally comparable relative increases in ethanol amounts in the suspensions from the strains WT 219 and ∆*pirC* 219. The photometric ethanol quantifications showed always substantial virtual ethanol amounts in the WT, whereas the GC quantification detected almost no ethanol in these cell suspensions. Moreover, the standard deviation among technical replicates was much higher for the enzymatic ethanol detections compared to the GC method. Finally, the photometric methods particularly the kit from the supplier r-biopharm Enzytec resulted in much higher ethanol contents than the direct ethanol quantification via GC (Supplementary Figure S3). These results indicated that the GC method is the most reliable measure for ethanol quantification in cyanobacterial suspensions and was applied in the forthcoming experiments.

### Ethanol production experiments under different light conditions

The first ethanol production trials were performed at a light intensity of 250 μmol photons m^−2^ s^−1^ for 1 week. Cells of the wild type (WT) showed better growth than strains expressing the ethanol-producing cassette 219. The final cell density of the WT suspension was almost two-times higher than for the “ethanol-producing” strains (Supplementary Figure S4A). Growth rates appeared slower after day 3, which might indicate an increasing light limitation.

**Figure 2:**
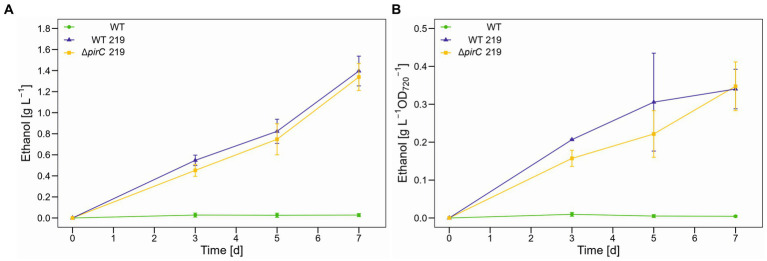
Ethanol production by different *Synechocystis* sp. PCC 6803 strains at 250 μmol photons m^−2^ s^−1^. Production assays were done in the Multicultivator MD1000 in BG11-Cu at high CO_2_ (5%, HC). **(A)** Ethanol titer per culture volume. **(B)** Ethanol titer per biomass as OD_720_. Mean values and standard deviations are shown (WT: *n* = 3; WT 219: *n* = 3; ∆*pirC* 219: *n* = 4).

Using the GC quantification, the ethanol production showed similar increase in the WT background as in the ∆*pirC* mutant (Figure 2), whereas the suspension of WT or ∆*pirC* mutant strain without cassette 219 remained virtually “ethanol free” when cultivated at 250 μmol photons m^−2^ s^−1^ for 1 week. The final ethanol titer of strains WT 219 and ∆*pirC* 219 reached 1.5 g L^−1^. When ethanol production was normalized to optical density, the ethanol accumulation appears to be slower in strain ∆*pirC* 219 during the first 5 days, whereas after 7 days similar ethanol values per optical density were achieved in both ethanol-producing strains (Figure 2B).

The faster growth of the WT compared to strain WT 219 indicated that the ethanol production induced a significant burden. To rule out the possibility that light might be the limiting factor for ethanol production, experiments were conducted at two-times higher light fluence rates, i.e., 500 μmol photons m^−2^ s^−1^. As expected, the higher light intensity permitted better growth of the ethanol-producing strains. The WT grew faster in the beginning, but reached a plateau after 3 days, whereas the ethanol-producing strains continued to increase their cell density until day 7, when all cultures showed almost similar optical densities (Supplementary Figure S4B).

**Figure 3:**
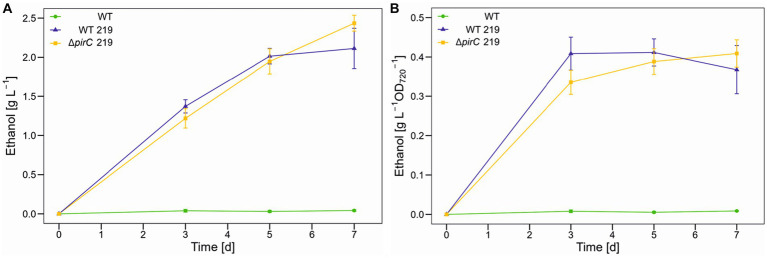
Ethanol production by different *Synechocystis* sp. PCC 6803 strains at 500 μmol photons m^−2^ s^−1^. Production assays were done in the Multicultivator MD1000 in BG11-Cu at high CO_2_ (5%, HC). **(A)** Ethanol titer per culture volume. **(B)** Ethanol titer per biomass as OD_720_. Mean values and standard deviations are shown (*n* = 2).

The higher light availability had also a positive impact on ethanol productivity, which increased by 66% to 2.5 g L^−1^ (Figure 3A). However, despite the further increase in biomass, a plateau of ethanol per biomass was reached after 3 days (Figure 3B). The ethanol production in the strain ∆*pirC* 219 seems to be higher than for strain WT 219 at day 7. This could indicate that the *pirC* mutation only resulted in higher ethanol titers after long-term cultivation. Therefore, the experiment was repeated and sampling time was extended up to 14 days. However, the ethanol production did not increase to significantly higher levels and was also not clearly different between WT 219 and ∆*pirC* 219 (data not shown).

### Ethanol production experiments under lowered nitrogen supply

**Figure 4:**
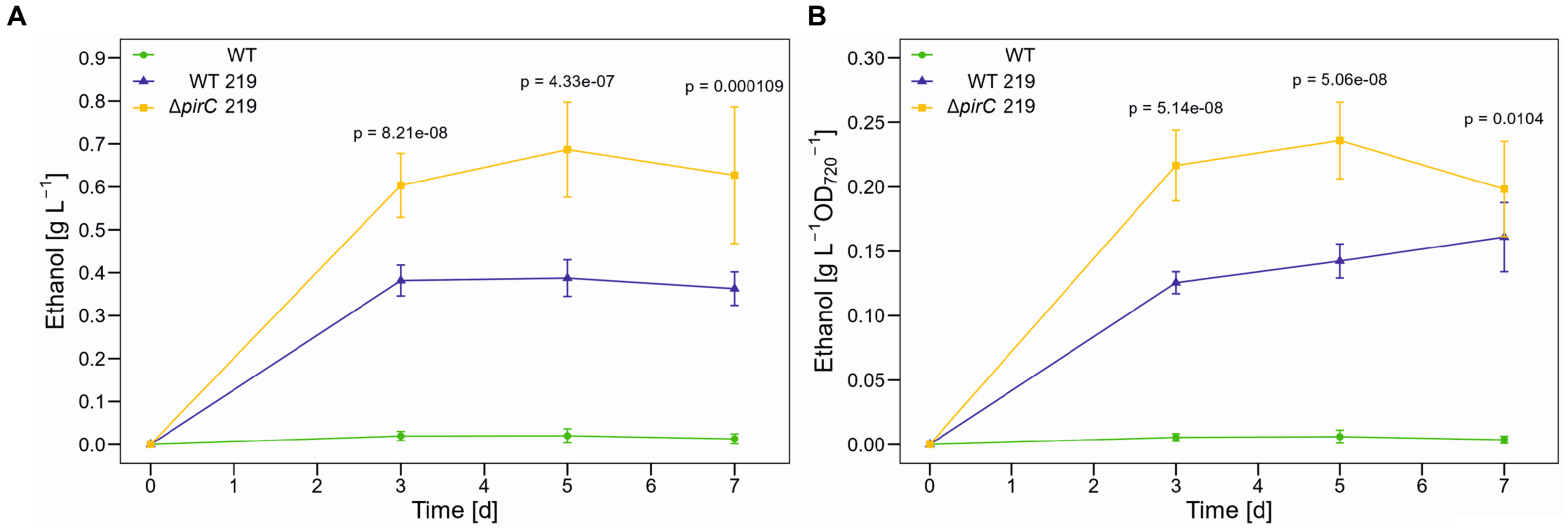
Ethanol production by different *Synechocystis* sp. PCC 6803 strains at 500 μmol photons m^−2^ s^−1^ and reduced nitrogen content. Production assays were done in the Multicultivator MD1000 in BG11-Cu with 1.8 mM NaNO_3_ (1/10 nitrate of the original BG11 medium) at high CO_2_ (5%, HC). **(A)** Ethanol titer per culture volume. **(B)** Ethanol titer per biomass as OD_720_. Mean values and standard deviations are shown (*n* = 4). Statistically significant differences between WT 219 and *∆pirC* 219 are indicated with the respective *p*-values (significant when *p* ≤ 0.05).

Because the highest impact of the *pirC* mutation on pyruvate pool size was initially observed under N-limiting conditions (Orthwein et al., 2021), we conducted experiments in which high CO_2_ conditions were combined with lowered nitrogen, i.e., the cells were cultivated in BG11 medium with ten-times lowered nitrate at 500 μmol photons m^−2^ s^−1^. The lower nitrogen content had a marked impact on growth in all strains. After 3 days the increase in OD_720_ ceased (Supplementary Figure S4C). Compared to the growth in BG11 with full nitrate amounts, the final optical density was almost 50% lower in all cultures. The slower growth had also a negative overall impact on the ethanol production (Figure 4), which was more than 50% lower than in BG11 with full nitrate. However, in the presence of reduced N availability, the strain ∆*pirC* 219 produced two-times more ethanol than WT 219 per volume as well as per biomass.

### Impact of ethanol production on the overall metabolism

**Figure 5:**
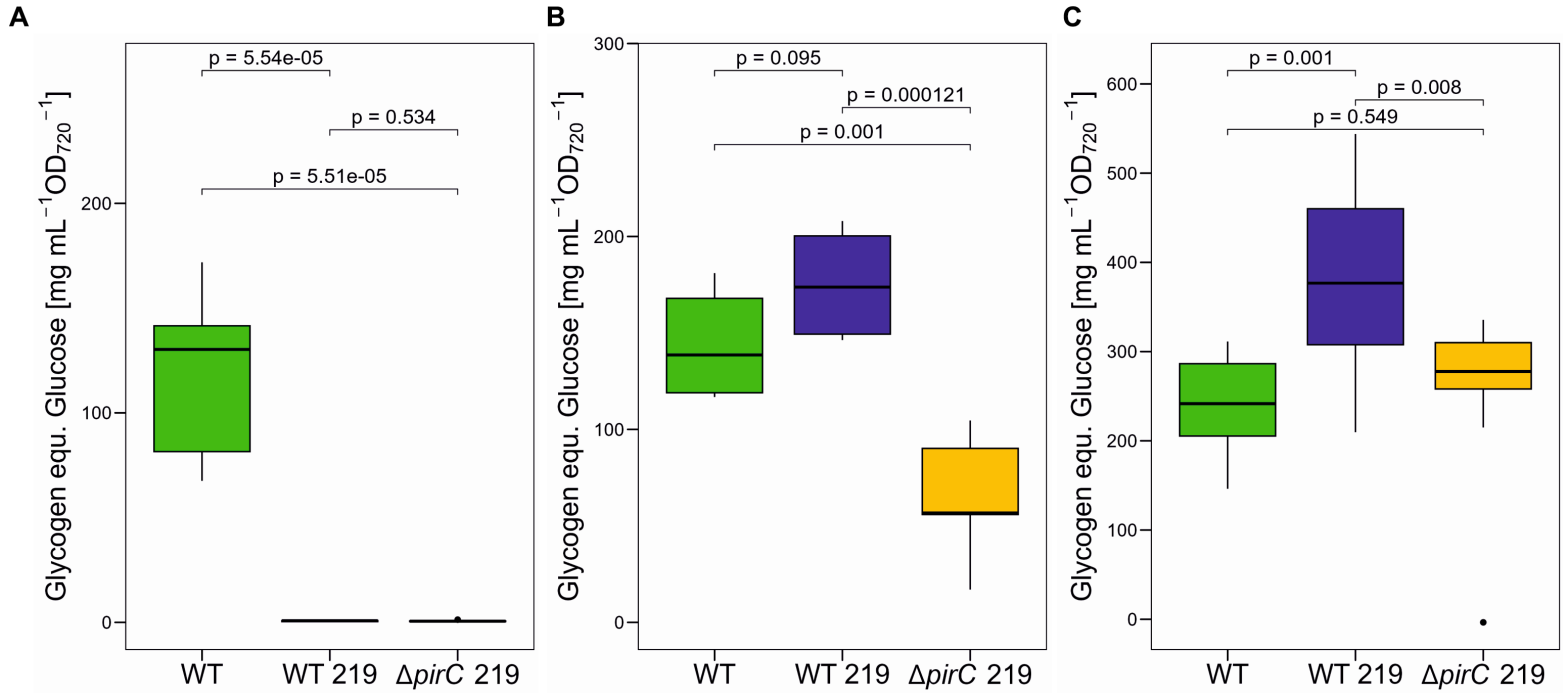
Glycogen accumulation in different *Synechocystis* sp. PCC 6803 strains. The strains were cultivated in BG11-Cu at 250 μmol photons m^−2^ s^−1^ (WT: *n* = 3; WT 219: *n* = 3; ∆*pirC* 219: *n* = 4) **(A)**, 500 μmol photons m^−2^ s^−1^ (WT: *n* = 2; WT 219: *n* = 2; ∆*pirC* 219: *n* = 2) **(B)**, or at 500 μmol photons m^−2^ s^−1^ in BG11-Cu with 1.8 mM NaNO_3_ (1/10 nitrate of the original BG11 medium, WT, *n* = 4, WT 219, *n* = 4, ∆*pirC* 219, *n* = 4) **(C)** for 7 days. Glycogen was quantified as glucose released by α-amylase from the cell pellets. Mean values and standard deviations are shown (*n* = 3). Statistically significant differences are indicated with the respective *p*-values (significant when *p* ≤ 0.05).

In addition to ethanol, we also quantified several key metabolites to get an impression about the impact of ethanol production on the entire carbon and nitrogen metabolism in the different strains. Glycogen represents the major high molecular mass carbon storage and becomes particularly accumulated under excess carbon supply, i.e., under high CO_2_ conditions (Eisenhut et al., 2007) or after strong nitrogen limitations (Doello et al., 2022). When grown at 250 μmol photons m^−2^ s^−1^, only cells of the WT accumulated substantial amounts of glycogen, whereas the ethanol-producing strains WT 219 and ∆*pirC* 219 were virtually free of glycogen (Figure 5A). This finding indicates that a substantial amount of fixed organic carbon is drained into ethanol and no longer stored as glycogen. The picture changed, when the light intensities was increased to 500 μmol photons m^−2^ s^−1^. Under this condition, WT and WT 219 accumulated similarly high amounts of glycogen, whereas the strain ∆*pirC* 219 accumulated only 47% of the glycogen amount compared to WT (Figure 5B). This finding is consistent with the notion that the *pirC* mutation directs organic carbon more towards lower glycolysis than in the direction of carbon storage (Orthwein et al., 2021). As expected, the decrease of the nitrate content in BG11 to one-tenth stimulated the glycogen accumulation in all strains compared to the cultivation at the same light intensity with standard BG11 (Figures 5B,C). The highest increase was observed in cells of the strain WT 219 (Figure 5C). Collectively, these data show that only under lower light the ethanol production is successfully competing with glycogen accumulation, while the *pirC* mutation decreases glycogen accumulation at high light in the BG11 with normal nitrate amounts, which however had no positive impact on the ethanol titer (see Figure 3).

**Figure 6:**
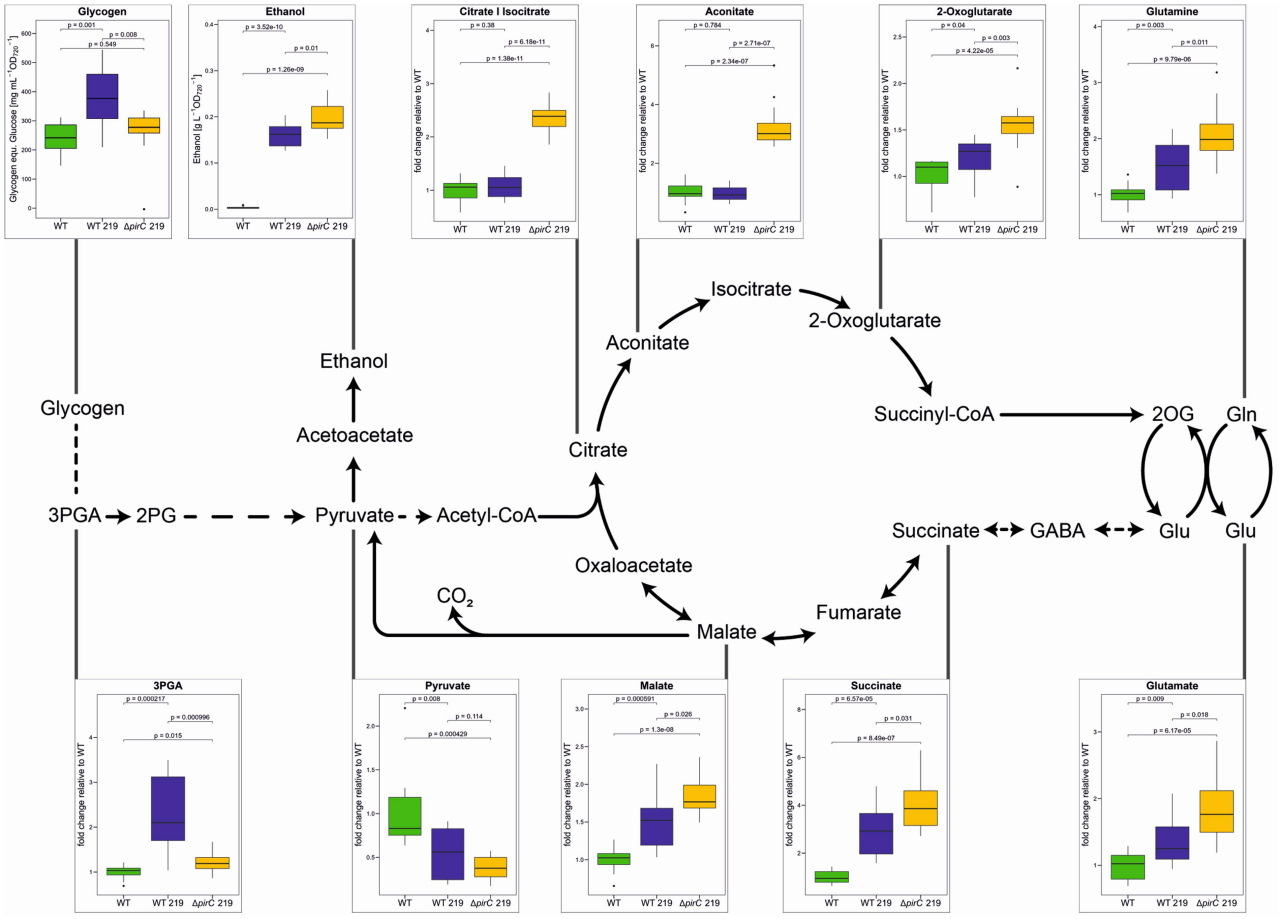
Metabolic changes of different *Synechocystis* sp. PCC 6803 strains during ethanol production. The strains were cultivated at 500 μmol photons m^−2^ s^−1^ in BG11-Cu with 1.8 mM NaNO_3_ (1/10 nitrate of the original BG11 medium) for 7 days. With the exception of glycogen and ethanol, all metabolites were quantified by LC/MS-MS. Mean values and standard deviations are shown (*n* = 4). Statistically significant differences are indicated with the respective *p*-values (significant when *p* ≤ 0.05).

Furthermore, targeted metabolome analysis using LC/MS-MS was applied to characterize changes in the steady state values of primary carbon and nitrogen metabolism intermediates. The method permitted to quantify most amino acids, several organic acids as well as 3-phosphoglycerate (3PGA), the primary CO_2_-fixing product of Rubisco in the Calvin cycle. Metabolite levels related to the primary carbon and nitrogen metabolism relevant for ethanol production are displayed for the strains under our three experimental conditions (Figure 6; Supplementary Figure S5). Under all conditions, higher steady state values of many intermediates in the oxidative branch of tricarboxylic acid (TCA) cycle such as citrate/isocitrate, aconitate, and 2-oxoglutarate were detected in the strain ∆*pirC* 219 compared to WT. These findings are consistent with the assumption that the mutation of *pirC* stimulates the carbon export from the Calvin cycle into lower glycolysis and subsequently into the TCA cycle. Malate and succinate, intermediates of the reductive branch of the TCA cycle, were also elevated in strain ∆*pirC* 219 and to a lesser extent in WT 219 compared to the WT (Figure 6), which might indicate an increased closure of the “open” TCA cycle under ethanol-producing conditions.

Regarding the ethanol precursor, pyruvate, always lower amounts were detected in the ethanol-producing strains compared to WT. When the strains were grown at the lowest light intensity of 250 μmol photons m^−2^ s^−1^, almost no pyruvate remained detectable in the strains WT 219 and ∆*pirC* 219, whereas some pyruvate was observed in these strains at the high light intensity of 500 μmol photons m^−2^ s^−1^ (Figure 6; Supplementary Figure S5). A similar picture appeared for 3PGA, which was near the detection limit at 250 μmol photons m^−2^ s^−1^ in the ethanol-producing strains. Together with the virtual absence of glycogen at 250 μmol photons m^−2^ s^−1^ (Figure 5), these findings indicate that CO_2_ fixation is light-limited in the ethanol-producing strains, which is overcome at 500 μmol photons m^−2^ s^−1^. Interestingly, lower 3PGA levels were detected when cells were incubated at 500 μmol photons m^−2^ s^−1^ in BG11 with reduced nitrate in ∆*pirC* 219 compared to WT 219 (Figure 6). This observation correlates with the enhanced ethanol production under this condition by strain ∆*pirC* 219 and indicates an increased export of 3PGA into lower glycolysis and ethanol in the absence of the carbon regulator *pirC*. Moreover, increased glutamate levels are present in the ethanol-producing strains compared to WT under all conditions. Glutamate increased four-fold compared to WT at 500 μmol photons m^−2^ s^−1^ in BG11 with standard nitrate amount, whereas it accumulated only two-fold in strain ∆*pirC* 219 and 1.4-fold in WT 219, respectively, in the presence of one-tenth nitrate (Figure 6; Supplementary Figure S5B). Similar relative increases were observed for 2-oxoglutarate and glutamine in the ethanol-producing strains compared to WT when cultivated at reduced nitrate amounts (Figure 6).

## Discussion

Collectively, our results show that a direct correlation between the rate of ethanol production and growth exists in our engineered strains. The productivity was stimulated by high CO_2_ and increasing light intensities. At moderate light of 250 μmol photons m^−2^ s^−1^, the ethanol production was clearly light-limited and presented a clear metabolic burden, because the ethanol-producing strains grew much slower than the WT. Furthermore, the decrease of growth due to limited nitrogen supply had also a rather negative impact on ethanol production. The strains WT 219 and Δ*pirC* 219 showed the highest ethanol titers per culture volume as well as per biomass when grown at high CO_2_ in standard BG11-Cu with 500 μmol photons m^−2^ s^−1^. After 7 days, an ethanol concentration of 2.43 g L^−1^ was detected for Δ*pirC* 219 and 2.11 g L^−1^ for WT 219. The total ethanol production is certainly higher than estimated from the cultures, because part of the ethanol can evaporate from the cell suspension due to its volatility. The quantification of these losses revealed that about 20% of the ethanol is evaporating from the cultures due to aeration (Supplementary Figure S6).

The first reported ethanol production upon engineering the cyanobacterial strain *Synechococcus elongatus* PCC 7942 resulted in the production of 1.5 mM (0.07 g L^−1^) ethanol within 3 weeks (Deng and Coleman, 1999). Later on, most ethanol-producing attempts were done using the strain *Synechocystis* sp. PCC 6803. Our maximum ethanol concentrations are clearly higher than the 0.46 g L^−1^, which were achieved when *Synechocystis* sp. PCC 6803 was genetically modified for the first time to produce ethanol (Dexter and Fu, 2009), which is likely due to the use of the NADPH-dependent ADH from *Synechocystis* sp. PCC 6803 in our study. In another study, a *Synechocystis* sp. PCC 6803 strain with knocked out pathways for polyhydroxybutyrate (PHB) and glycogen (Δ*glg* Δ*phaCE*/EtOH) syntheses produced 2.96 g L^−1^ after 3 days in very dense cultures at an OD_730_ of 50 (Namakoshi et al., 2016), which is similar to the ethanol amounts reported here. An increased ethanol production was also observed by stimulating the CO_2_ fixation in *Synechocystis* sp. PCC 6803 (Roussou et al., 2021). The expression of a second copy of the ethanologenic cassette in a PHB mutant background resulted in 5.5 g L^−1^ after a cultivation period of 26 days (Gao et al., 2012). In the latter study, the absolute ethanol titer after more than 3 weeks was thus higher than in our study, but it is again in the order of magnitude of 2–3 g L^−1^ per week that was reported here or in the other mentioned studies. Unfortunately, a direct comparison of the results is only possible to a limited extent, as the experimental conditions are not comparable with regard to culture density, cultivation duration, illumination intensity, etc. A negative impact of the produced ethanol on the cyanobacterial strains can be excluded. It has been shown that metabolically active and productive cultures can be maintained over a longer period of time in the presence of up to 15 g L^−1^ ethanol, whereas cultures with 20 g L^−1^ ethanol showed significantly slower growth (Lopes da Silva et al., 2018).

Our main aim was to prove, whether or not the mutation of *pirC* has a positive impact on ethanol production. Previous studies revealed that the available amount of pyruvate seems to be the bottleneck for ethanol production (Kopka et al., 2017), while mutation of *pirC* increased the internal pyruvate pool at least under nitrogen-limiting conditions (Orthwein et al., 2021). Hence, mutation of *pirC* and thereby stimulation of carbon flow into lower glycolysis seemed to be a promising strategy to improve ethanol production in cyanobacteria as has been also proposed by Andrews et al. (2021). Our results revealed that a positive impact of *pirC* mutation was only visible under nitrogen-limiting conditions, whereas in BG11 medium with full nitrate concentration only a slight stimulation of ethanol production was observed in the strain Δ*pirC* 219 compared to WT 219. This result was not expected, since we initially assumed that the absence of *pirC* could also stimulate carbon export into lower glycolysis and thus support ethanol synthesis under normal nitrogen availability. Indeed, our metabolic investigations showed slightly increased pyruvate levels in strain Δ*pirC* 219 when grown at high CO_2_ with 500 μmol photons m^−2^ s^−1^. However, the strain accumulated still substantial amounts of glycogen under this condition, which probably prohibited a higher stimulation of ethanol production in strain Δ*pirC* 219 compared to WT 219. This assumption is supported by the experiments at high light (500 μmol photons m^−2^ s^−1^) and BG11 with ten-times reduced nitrate content. Only under this condition a clear advantage of the *pirC* mutation on ethanol production was observed, which is correlating with the enhanced metabolite levels in the oxidative branch of the TCA cycle in strain Δ*pirC* 219. This observation suggests that the mutation of *pirC* is accompanied by the expected stimulation of carbon export from the Calvin cycle into lower glycolysis and subsequently into the TCA cycle. However, if the measured changes in steady state metabolite levels indeed represent elevated carbon flux into lower glycolysis including the ethanol precursor pyruvate needs to be addressed by 13C-labelling analysis in the future. This could be further supported by comparing the acetyl-CoA levels in ethanol-producing strains with WT, which was for technical reasons not possible with our LC-MS/MS method.

Collectively our results indicate that the redirection of carbon into lower glycolysis due to mutation of *pirC* is obviously not sufficient to enhance the carbon partitioning into ethanol under high nitrate availability to promote faster growth. In future attempts, the *pirC* mutation should be combined with other measures such as the successful effects of glycogen and PHB synthesis knock outs. Moreover, a previous study showed a rather moderate, only up to fourfold higher expression of the *adh* gene under copper-free conditions applying the same ethanologenic cassette (Dienst et al., 2014). Therefore, we suggest that a higher expression of the “ethanol-producing” genes might also have a further stimulatory effect, because there is still a substantial pyruvate pool left and many intermediates of the TCA cycle are elevated in the *pirC* mutant background. These metabolic features indicate that the carbon drain from pyruvate into the direction of ethanol is not strong enough to efficiently use the photosynthetically produced organic carbon for ethanol production.

## Data availability statement

The original contributions presented in the study are included in the article/Supplementary material, further inquiries can be directed to the corresponding author.

## Author contributions

MH designed the study and wrote manuscript with contributions from all coauthors. JB, KK, KM, and E-MB performed experiments. LE estimated and calculated ethanol via GC. MH and E-MB supervised experiments and evaluated data. All authors contributed to the article and approved the submitted version.

## Funding

The PostDoc position of E-MB is funded by the EU Consortium Gain4Crops (grant to MH, No. 862087). The work at the University Rostock on metabolic regulation of the carbon metabolism in cyanobacteria is performed in the Research Consortium SCyCode (FOR 2816) that is supported by the German Research Foundation (DFG, HA2002/23-2).

## Conflict of interest

The authors declare that the research was conducted in the absence of any commercial or financial relationships that could be construed as a potential conflict of interest.

## Publisher’s note

All claims expressed in this article are solely those of the authors and do not necessarily represent those of their affiliated organizations, or those of the publisher, the editors and the reviewers. Any product that may be evaluated in this article, or claim that may be made by its manufacturer, is not guaranteed or endorsed by the publisher.
