# Supplementary material for: Impact of the carbon flux regulator protein pirC on ethanol production in engineered cyanobacteria
Source: Front Microbiol. 2023 Aug 15;14:1238737. doi: 10.3389/fmicb.2023.1238737 (PMC10465007; doi:10.3389/fmicb.2023.1238737)
Supplement: Supplementary file 1 [file Data_Sheet_1.pdf]

Supplementary Material for the Manuscript

Impact of the carbon flux regulator protein PirC on ethanol production in engineered cyanobacteria

Julien Böhm[1,2], Karsten Kauss[1], Klaudia Michl[1], Lisa Engelhardt[3], Eva-Maria Brouwer[1], Martin Hagemann[1]*

Institute of Biosciences, (1) Department of Plant Physiology, (2) Department Aquatic Ecology, (3) Department Microbiology, University of Rostock, Rostock, Germany

***Corresponding author:** Martin Hagemann, Institut für Biowissenschaften, Abteilung Pflanzenphysiologie, Universität Rostock, A.-Einstein-Str. 3, Rostock D-18059, Germany; Tel: +49(0)3814986110; Fax: +49(0)3814986112; Email: martin.hagemann@uni-rostock.de; http://orcid.org/0000-0002-2059-2061

Supplementary Material

Table S1: Strains used and generated in the present study
Table S2: Primers used in the present study

Figure S1: Genotyping of the investigated *Synechocystis* sp. PCC 6803 strains via PCR
Figure S2: Ethanol production under N-free conditions in different strains of *Synechocystis*
Figure S3: Comparison of different ethanol quantification methods
Figure S4: Growth of different strains of *Synechocystis* during ethanol production experiments
Figure S5: Metabolic changes of different strains of *Synechocystis* during ethanol production
Figure S6: Quantification of ethanol losses with the gas stream

Supplementary Tables

Supplementary Table S1: Strains used and generated in the present study

Organisms	Strain	Genotype
Synechocystis sp. PCC 6803 (glucose-sensitive)	Wild type (WT)	Wild type
Synechocystis sp. PCC 6803 with the ethanologenic cassette 219	WT 219	pVZ325-P_{petJ}-PDC-synADH-$Gent^R$
Synechocystis sp. PCC 6803 mutant Δ*pirC*	Δ*pirC*	*pirC*::Kan^R
Synechocystis sp. PCC 6803 mutant Δ*pirC* with the ethanologenic cassette 219	Δ*pirC* 219	*pirC*::Kan^R pVZ325-P_{petJ}-PDC-synADH-$Gent^R$
E. coli/ DH5α wild type	DH5α	F^- φ80*lac*ZΔM15 Δ(*lac*ZYA-*arg*F)U169 *rec*A1 *end*A1 *hsd*R17(r_K^-, m_K^+) *pho*A *sup*E44 λ^-*thi*-1 *gyr*A96 *rel*A1
E. coli/ DH5α with the ethanologenic cassette 219	DH5α 219	pVZ325-P_{petJ}-PDC-synADH-$Gent^R$
E. coli	DH5α RP4	RP4 plasmid

Supplementary Table S2: Primers used in the present study

Name	Sequence (5‘-3‘)	Company
PDC_intern_fw	AATCGCCCGTCAGGTCGAAG	Operon
PDC_intern_rev	GATTCCGCCGTGGCACAAAG	Operon
synADH_intern_fw	GTGTGCCACAGTGATTTGTC	Operon
synADH_intern_rev	GTTCCACCACGGGTTTAATG	Operon
sll0944(pirC)_for	GTGCAATGCCTCGGAAAAATTT	Microsynth
sll0944(pirC)_rev	CTAAGCCACTAAGGATTGGGAAG	Microsynth
6803 pirCKOcheck_fw	TGGCATGGCCTAAGTATTCC	Microsynth
6803 pirCKOcheck_rev	GCGTTCTGCAGGGGATTACC	Microsynth

Supplementary Figures

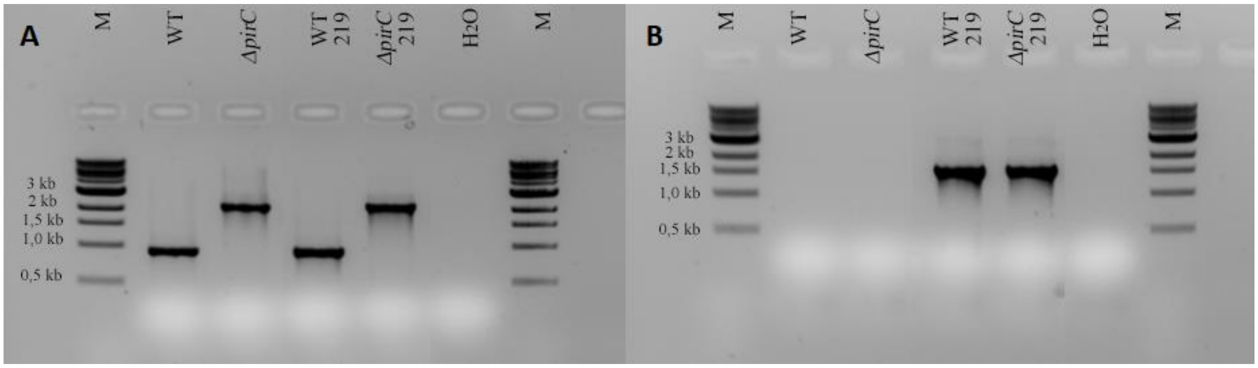

Suppl. Figure S1: Genotyping of the investigated *Synechocystis* sp. PCC 6803 strains via PCR. (A) Verification of the *pirC* mutation in the mutant Δ*pirC* and the strain Δ*pirC* 219 using the primer pair 6803 pirCKOcheck_fw and 6803 pirCKOcheck_rev. Please note that the larger fragment in the strains with inactivated *pirC* gene is due to Km^R cassette insertion. **(B)** Occurrence of the ethanologenic cassette in the strains Δ*pirC* 219 and WT 219 using the primer pair PDC_intern_fw and synADH_intern_rev. Primer sequences are given in the Suppl. Table S2. (WT: wild type; M: DNA size marker; H_2O: negative control without DNA)

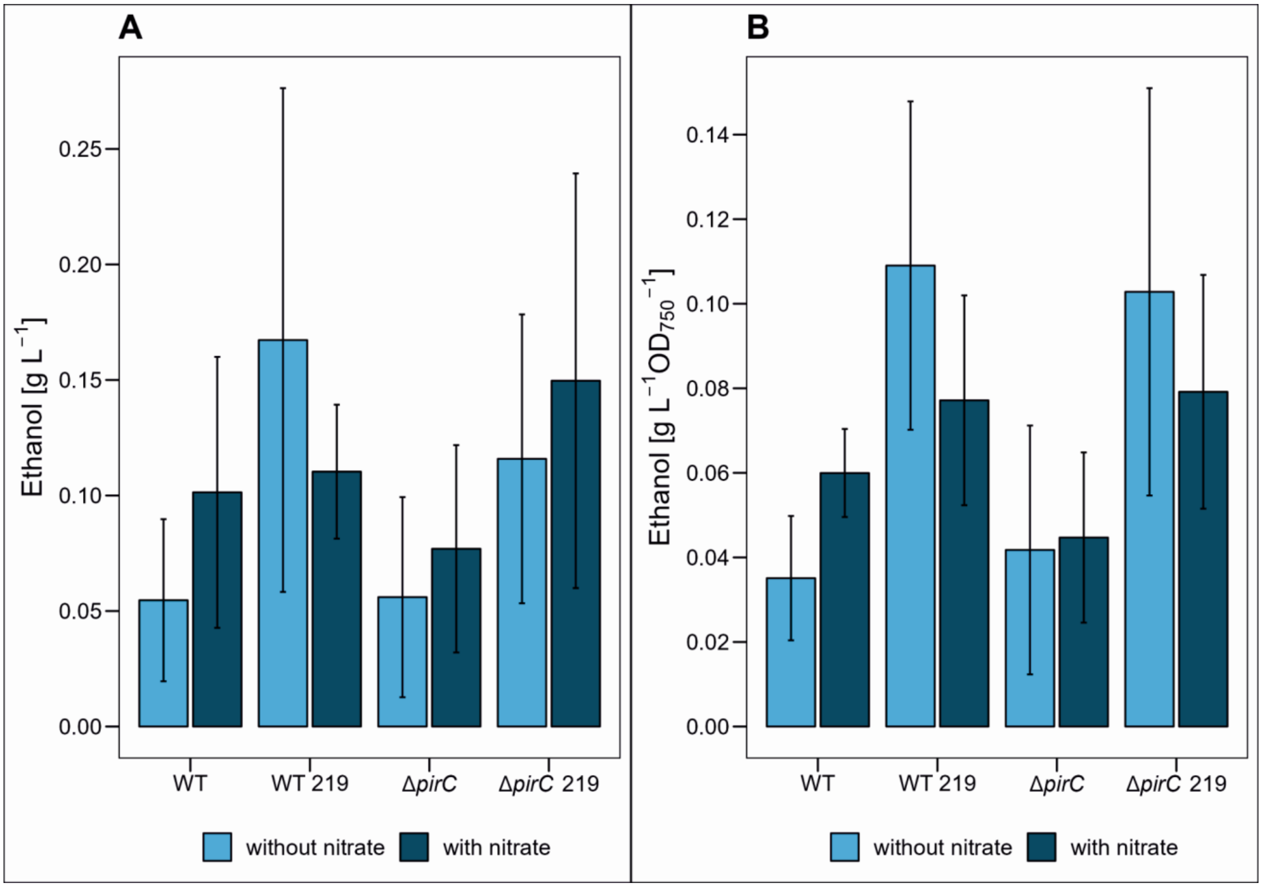

Suppl. Figure S2: Ethanol production under N-free conditions in different strains of *Synechocystis* sp. PCC 6803. Cells were grown in shaken Erlenmeyer flasks for up to 16 days in complete or nitrate-free BG11 medium. Ethanol values are shown per culture volume **(A)** or normalized to biomass **(B)**. The data represent mean values with standard deviation from three independent cultivation experiments.

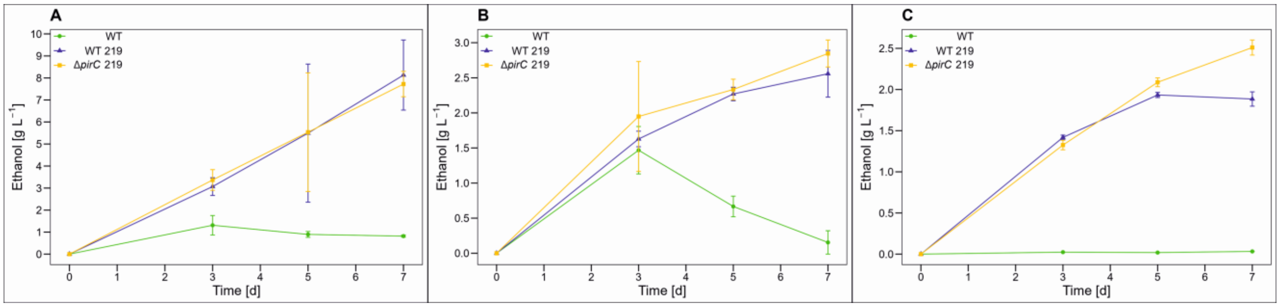

Suppl. Figure S3: Comparison of different ethanol quantification methods. Ethanol was quantified in identical cultures of different strains of *Synechocystis* sp. PCC 6803 (WT: wild type; WT 219: wild type with ethanologenic cassette; Δ*pirC* 219: mutant Δ*pirC* with ethanologenic cassette). **(A)** Kit from r-biopharm Enzytec; **(B)** Ethanol Assay Kit (ab272531) abcam; **(C)** gas chromatography.

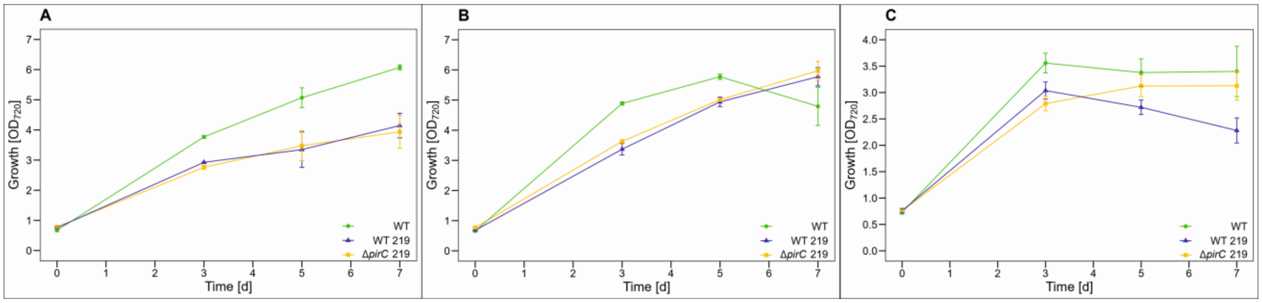

Suppl. Figure S4: Growth of different strains of *Synechocystis* sp. PCC 6803 during ethanol production experiments. Production assays were done in the Multi-Cultivator MD1000 in BG11 –Cu at high CO_2 (5%, HC). Due to the high culture density, OD_{720} was determined manually using diluted suspensions at a photometer. **(A)** 250 µmol photons m^{-2} s^{-1} (n=3); **(B)** 500 µmol photons m^{-2} s^{-1} (n=2); **(C)** 500 µmol photons m^{-2} s^{-1} in BG11-Cu with ten-fold lowered nitrate content (n=4). Mean values and standard deviations are shown.

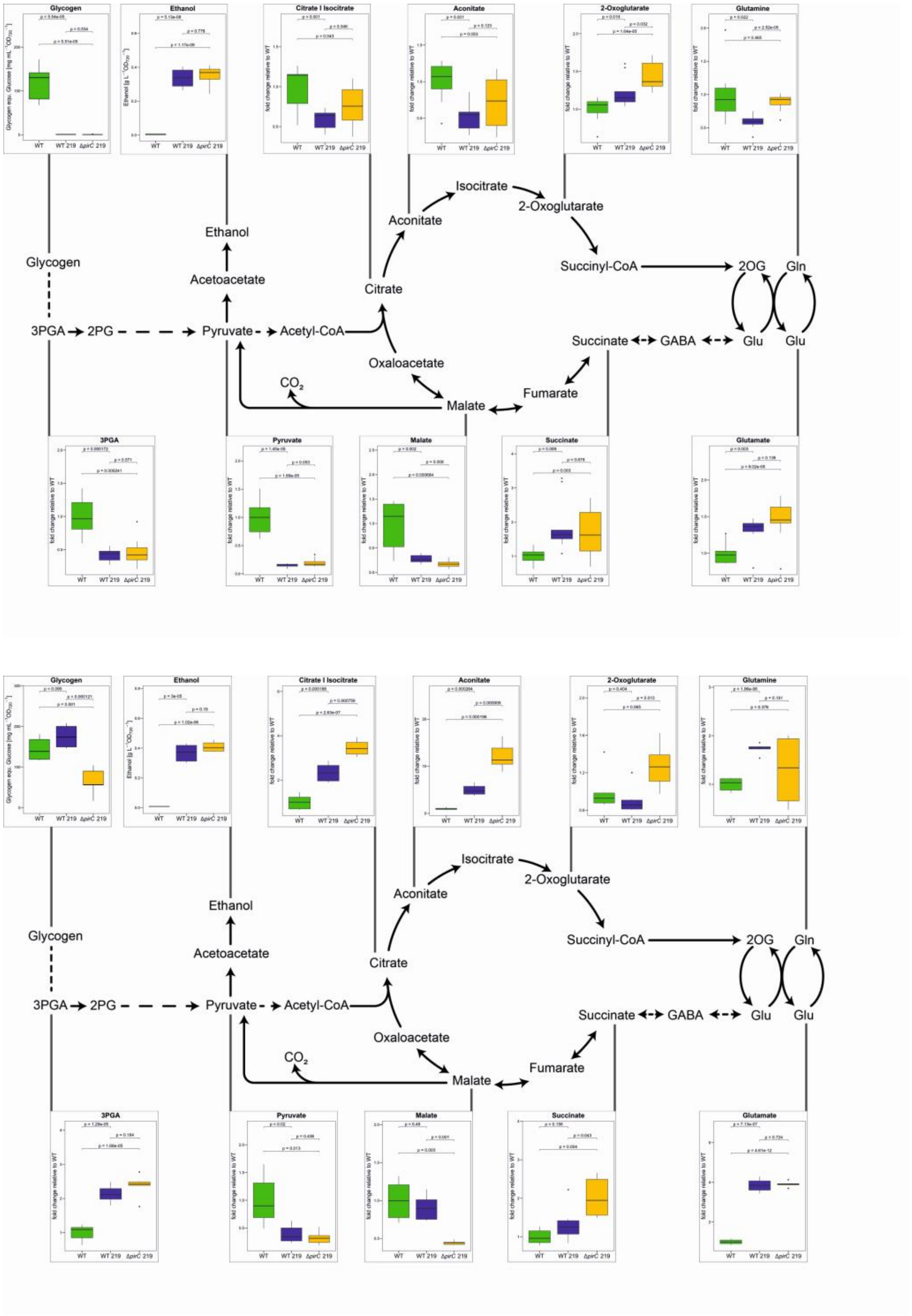

Suppl. Figure S4: Metabolic changes of different strains of *Synechocystis* sp. PCC 6803 during ethanol production. The strains were cultivated at 250 µmol photons m^{-2} s^{-1} (n=3; **upper panel**) or 500 µmol photons m^{-2} s^{-1} (n=2; **lower panel**) in BG11-Cu for 7 days. With the exception of glycogen and ethanol, all metabolites were quantified by LC/MS-MS. Mean values and standard deviations are shown. Statistical significant differences are indicated with the respective p values (significant when $p \leq 0.05$).

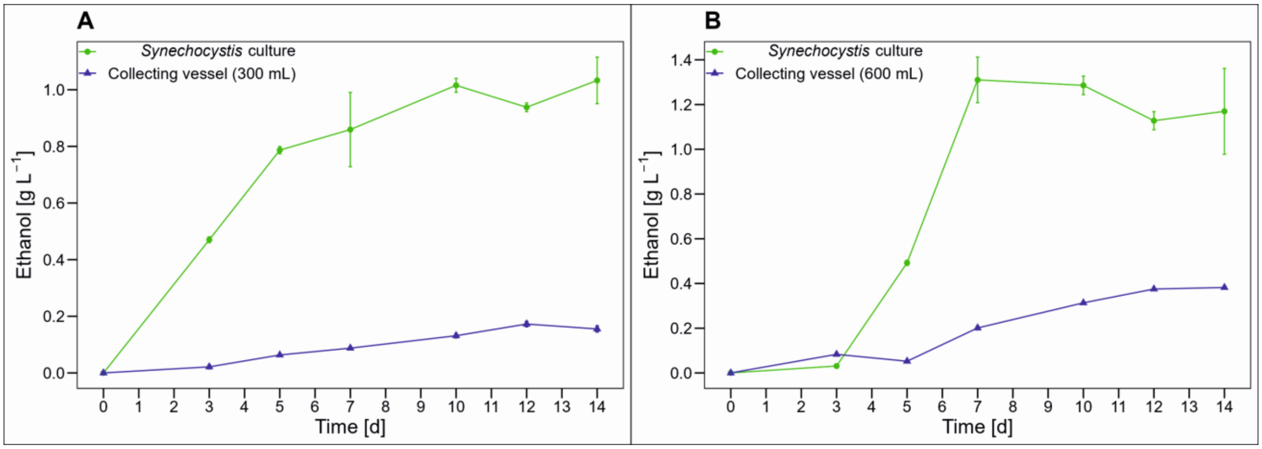

Suppl. Figure S6: Quantification of ethanol losses with the gas stream. The gas stream leaving the ethanol-producing cyanobacterial cultures was flushed through bottles containing either 300 **(A)** or 600 **(B)** mL distilled water. The ethanol content in the collection vessel was normalized to the volume of the cultivation vessel. The total ethanol amounts produced in the cultures (solid lines) or dissolved in the bottles (broken lines) were compared over 14 days.
